# Molecular Diagnostics and Genetic Counseling in Primary Congenital Glaucoma

**DOI:** 10.5005/jp-journals-10008-1133

**Published:** 2013-01-15

**Authors:** Muneeb Faiq, Kuldeep Mohanty, Rima Dada, Tanuj Dada

**Affiliations:** Laboratory for Molecular Reproduction and Genetics, Department of Anatomy, All India Institute of Medical Sciences New Delhi, India; Laboratory for Molecular Reproduction and Genetics, Department of Anatomy, All India Institute of Medical Sciences New Delhi, India; Laboratory for Molecular Reproduction and Genetics, Department of Anatomy, All India Institute of Medical Sciences New Delhi, India; Glaucoma Services, Dr RP Centre for Ophthalmic Sciences, All India Institute of Medical Sciences, New Delhi, India

**Keywords:** Primary congenital glaucoma, Molecular diagnostics, Genetics, Genetic counseling.

## Abstract

Primary congenital glaucoma (PCG) is a childhood irreversible blinding disorder with onset at birth or in the first year of life. It is characterized by the classical traid of symptoms *viz*. epiphora (excessive tearing), photophobia (hypersensitivity to light) and blepharospasm (inflammation of eyelids). The only anatomical defect seen in PCG is trabecular meshwork dysgenesis. PCG shows autosomal recessive mode of inheritance with considerable number of sporadic cases. The etiology of this disease has not been fully understood but some genes like CYP1B1, MYOC, FOXC1, LTBP2 have been implicated. Various chromosomal aberrations and mutations in mitochondrial genome have also been reported. Molecular biology has developed novel techniques in order to do genetic and biochemical characterization of many genetic disorders including PCG. Techniques like polymerase chain reaction, single strand conformational polymorphism and sequencing are already in use for diagnosis of PCG and other techniques like protein truncation testing and functional genomics are beginning to find their way into molecular workout of this disorder. In the light of its genetic etiology, it is important to develop methods for genetic counseling for the patients and their families so as to bring down its incidence. In this review, we ought to develop a genetic insight into PCG with possible use of molecular biology and functional genomics in understanding the disease etiology, pathogenesis, pathology and mechanism of inheritance. We will also discuss the possibilities and use of genetic counseling in this disease.

**How to cite this article:** Faiq M, Mohanty K, Dada R, Dada T. Molecular Diagnostics and Genetic Counseling in Primary Congenital Glaucoma. J Current Glau Prac 2013;7(1):25-35.

## INTRODUCTION

Primary congenital glaucoma (PCG) is an idiopathic, infantile, irreversibly blinding disorder characterized by progressive loss of retinal ganglion cells with a characteristic hallmark of trabecular meshwork (TM) dysgenesis. It is a developmental malformation of the anterior chamber of the eye with early onset in life (within 1 year of birth). The principle mode of PCG inheritance is autosomal recessive. PCG accounts for 22.2% of all pediatric glaucoma cases. The onset of this disease is congenital or within the first year of birth with the classical triad of symptoms *viz* epiphora, photophobia and blepharospasm.^1^ This symptomatic picture arises due to corneal irritation and buphthalmos (eyeball enlargement) due to elevation in intraocular pressure (IOP). A total of 4.2% of all childhood blindness is due to PCG and it is the most common type (55%) of primary pediatric glaucomas.^2^ Pathologically PCG is an ocular malformation with dysgenesis of the TM and anterior chamber angle precipitating impediment to the aqueous humor dynamics consequently causing elevation in IOP (>12 mm Hg) which, in turn, leads to damage in optic nerve and, as a result, blindness.

### Need for Genetic Counseling in PCG

A diverse variation among many ethnic groups has been reported in the incidence of PCG ranging from as low as 1:10,000 in western populations^3^ to as high as 1:1, 250 in Slovakian gypsies. Its incidence is 1:2,500 in Saudi Arabia^4^ and 1:3, 300 in Andhra Pradesh state of India.^5^ Consanguinous marriages have been attributed as reasons behind high prevalence of PCG in many ethnic populations.^6-10^ The penetrance (the extent of manifestation of an inheritable trait) of PCG also varies from 40 to 100% with more males being affected than females.^9,10-14^ However, some studies suggest that there is equal prevalence of male and female cases. Some authors have reported familial pattern of PCG inheritance in 30 to 40% cases^15^ while other report only 11 to 14%.^10^ The genetic background of PCG, its mode of inheritance and need for genetic counseling is justified by three main points *viz*.

1. Genetic modes of PCG transmission have been studied and established in dogs.^16-18^
2. Correlation between PCG and consanguineous parents has been found to be significant.^6-10^
3. Siblings are affected in a very high number of cases.^3,6,7,10^

In this review we will discuss the genes involved in etiology and pathogenesis of PCG and the scope of many molecular biology techniques in the investigation of the molecular mechanisms and enhanced understanding of the disease. We will use this knowledge to see how it can be helpful in clinical setting for patient management, risk assessment and genetic counseling.

### Genetic Basis of PCG

Molecular genetics has greatly enhanced our understanding of the molecular basis of many inherited disorders. The chromosomal aberrations of a large number of genes cause physiological dysfunction. Not only this, the underlying genes and the pathogenic mutations thereof have been identified. This has led to the development of novel diagnostic avenues for previously idiopathic disorders. The technological advances in molecular diagnostics and genetics have aided us to characterize pathologic gene changes providing further insight into the molecular pathogenesis of these disorders. As a result of this, novel approaches toward diagnosis, management and prevention of a plethora of genetic conditions are emerging. In the present day circumstances, keeping pace of the rapid advances in genetic ophthalmology is a challenge to a clinician. Molecular genetic techniques have proved to be indispensible in many areas of inherited disorders including ophthalmic disease, such as PCG. There are two factors that shape the clinician's molecular approach toward a genetic disorder *viz* our understanding of the etiopathogenesis of the disease and the degree of complexity involved in its genetic hallmarks. Some diseases are caused by specific gene mutations where routine molecular diagnosis can be provided by a simple Polymerase chain reaction (PCR) based assay. But many diseases are caused by various mutations (known as well as unknown) and the lines of molecular diagnosis need to be well structured taking into account the size of the gene and its various regulatory regions. Well organized genetic workout is necessary in such cases otherwise the genetic diagnosis might be very costly without yielding any fruitful outcome. Location of mutations in the gene also has a role to play in structuring the molecular diagnosis and prognosis of a genetic disorder. If the mutation is located in exons, it might be easy to get it from mRNA from the tissues expressing this gene but if the mutations are present elsewhere then RNA-splicing, regulatory sequences upstream and downstream of the coding region pose difficulties for routine sequencing. It is understood that a very small percentage of genetic ophthalmic disorders can be treated efficiently; in the light of this fact, molecular diagnosis is becoming more and more imperative as it is likely to offer a lot of useful information for the clinician, patient and his/her family members. Here we will first discuss some of the genes, their mutations, chromosomal abnormalities and mitochondrial gene mutations involved in PCG and then talk about how molecular biology technology can help in identification, diagnosis and characterization of various pathological hallmarks of PCG.

**Table 1:** **Table 1:** Identifying various genetic loci involved in PCG

*Locus*		*Chromosomal location*		*Gene*		*References*	
GLC3A		2p21		CYP1B1		26	
GLC3B		1p36				27	
GLC3C		14q24.3-q31.1		LTBP2		28	

The etiopathogenesis of PCG is unknown but three chromosomal loci (GLC3A, GLC3B and GLC3C) have been identified for PCG. Table 1 gives the details of the mapped genetic loci of PCG with nomenclature of the loci (GLC acronym for glaucoma; numerals indicating type and alphabets the order of discovery). Table 1 also mentions names of the genes that have been identified till date. Among the loci identified GLC1A (myocilin), GLC1E (optineurin) for POAG and GLC3A (CYP1B1) for PCG have been characterized.^19-21^

**Fig. 1:**
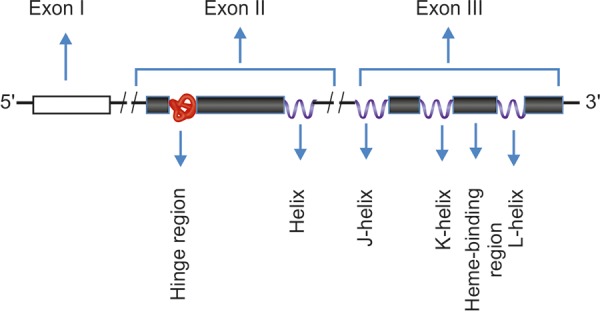
Schematic representation of CYP1B1 gene

### Genes involved in PCG

Cytochrome p450 1B1 (CYP1B1)

The Cytochrome p450 1B1 (CYP1B1) gene is located on chromosome 2 at position 2p21-22. It contains two introns and three exons and the open reading frame starts with exon II and ends within exon III. Figure 1 gives the schematic diagram of the CYP1B1 gene. The protein product of this gene metabolizes a variety of xenobiotics and endogenous intermediary substrates. It is expressed in the ciliary body, nonpigmented ciliary epithelium, iris and TM. CYP1B1 also produces molecular species which act across many signaling pathways thereby regulating expression of many genes involved in growth, development and differentiation of various ocular structures particularly those of the anterior chamber. It also plays a crucial role in the metabolism of retinol to all-trans-retinal and all-trans-retinoic acid.^22^ All-trans-retinoic acid is a powerful morphogen which regulates *in utero* development, growth and differentiation.^23^ Pathogenic mutations in CYP1B1 occur in PCG in varying frequencies; some are highly prevalent (e.g. Gly368Stop) and others appear with different frequencies (e.g. p.Gly252Arg, p.Gly367Arg and p.Pro370Leu) across population. As far as the current literature is concerned, mutations in CYP1B1 gene are the most common cause of PCG. Our research group has already reported novel mutations p.Ile94X, p.His279Asp, p.Gln340His, p.Lys433Lys^24^ p.Leu24Arg, p.Phe190Leu and p.Gly329Asp^25^ along with other mutations in CYP1B1 gene in PCG patients.

### Myocilin

**Fig. 2:**
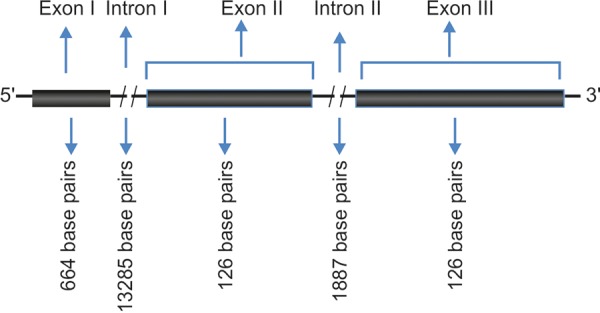
Fig. 2: Schematic representation of MYOC gene

MYOC (chromosome 1 at 1q25) codes for myocilin/TIGR (trabecular meshwork-induced glucocorticoid response) protein. MYOC gene consists of two introns and three exons. Figure 2 shows a schematic representation of the MYOC gene. Most tissues of the eye (trabecular meshwork, sclera, ciliary bodies, retina, etc.) express this protein.^29,30^ MYOC has been located in extracellular matrix of normal as well as glaucomatous trabecular meshwork. It codes for a sticky stress protein, which covers TM. Mutations in MYOC gene occur in a many types of eye disorders accounting for 2 to 5% cases of POAG^19^ and 5.5% of PCG cases.^31^ In our studies on MYOC gene analysis in PCG patients, we found five single nucleotide polymorphisms *viz* —126T>C, —83G>A, p.R76K, IVS2+35G>A and p.Y347Y.^32^

## FOXC1

**Fig. 3:**
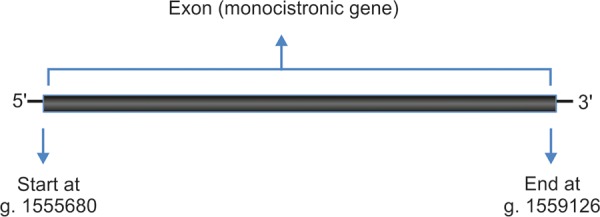
Schematic representation of FOXC1 gene

Another gene that is thought to play a role in the pathogenesis of PCG is Forkhead-related transcription factor C1 (FOXC1 or FKHL7). It is located on p-arm of chromosome 6 (6p25). A schematic representation of FOXC1 gene is given in Figure 3. Mutations in this gene have been found to be linked with PCG in many studies. Originally FOXC1 mutations were observed to be directly involved in eye conditions commonly and collectively referred to as anterior segment dysgenesis (ASD). Since, TM (tissue involved in elevation of IOP in case of PCG) is the part of the anterior segment, it forms a vindicated rationale to speculate its role in development and pathogenesis of PCG. We have reported two sequence variations *viz* GGC375ins and GGC447ins in FOXC1 gene in PCG patients tested negative for CYP1B1 mutations. However, we did not find any significant correlation between FOXC1 gene mutations and PCG.^32^

**Fig. 4:**
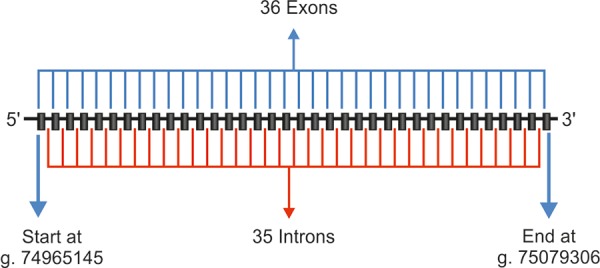
Schematic representation of LTBP2 gene

### LTBP2

Recently linkage analysis of PCG in consanguineous Pakistani PCG families was reported with involvement of a new chromosomal locus adjacent to GLC3C on 14q24.2-24.3.^33,34^ The candidate gene identified was latent transforming growth factor-b-binding protein 2 (LTBP2). Figure 4 shows a schematic diagram of the LTBP2 gene. Truncating mutation were identified in this gene in PCG patients.^35,36^ The expression pattern of LTBP2 in the trabecular meshwork, ciliary body and ciliary process^35^ has increased the complexity of the pathophysiological mechanism involved in PCG.

### Mitochondrial Mutations

A number of optic neuropathies like LHON and Wolframm syndrome are associated with mitochondrial abnormalities. In view of the fact that majority of cases for PCG still remain idiopathic and genetic abnormalities have been identified in only a small fraction of cases, it raises a very plausible possibility that mitochondrial mutations may play a role in pathogenesis of PCG induced by trabecular dysgenesis and consequent optic neuropathy. To bolster this, we have already reported a significant relation between mutations in mitochondrial genome and PCG.^37^ We have also proposed the mechanism of TM dysgenesis secondary to mitochondrial mutations.^37^

### Chromosomal Aberrations

In addition to gene and mitochondrial mutations, chromosomal abnormalities have also been reported in PCG. Examples of this include trisomy 8q22-qter, monosomy 9p23-qter, 22p+ variant chromosome, etc.^38-41^

### Rationale of Molecular Diagnostic Studies

The main mode of inheritance for PCG is autosomal recessive pattern with a few cases of pseudo-dominance and autosomal dominant inheritance. As a result of lack of access to tertiary care ophthalmic facilities, many PCG patients present at a late stage raising the propensity to increased risk of irreversible blindness. Early and reliable diagnosis is, therefore, extremely important to invoke suitable medical and surgical interventions which, in turn, can prevent loss of vision. Also early identification of mutations associated with a severe form of the disease as against those associated with milder form will aid in determining the management and prognosis. The molecular diagnosis technology may, in near future, find a place in identification of disease *in utero*^42^ via prenatal diagnosis in familial cases. This is likely to revolutionize the treatment strategies of PCG. Different mutations appear with different severity in the phenotype. Some mutations are associated with complete loss of function of the resultant protein and result in discrete clinopathological phenotype. For example frameshift and p.R390C homozygous mutations in CYP1B1 gene are associated with very severe disease phenotype and poor prognosis;^43^ while other mutations (e.g. G61E, R368H etc.) in which some residual activity of protein is still present, are associated with milder form of disease. Correlation of the genotype and phenotype will, for this reason, abet in better understanding of PCG etiopatho-genesis.

### Molecular Biology in Diagnosis of PCG

Molecular diagnosis technology provides help to the patient and his/her family by bringing down the prevalence of inherited disorders in a population as well as across generations. It affects the patient as well as the other family members and is a very important component in understanding many genetic disorders. Well structured and informed counseling gives a foundation for correct decision making to the patients and their families. While counseling, patients need to be informed regarding clinical features of their disease and its effects on the family. Patient and family also need to be informed about some important genetic factors including the mode of inheritance and risk.

Since many genes causing PCG have been identified (while others remain elusive), molecular diagnosis technology can be performed by mutational analysis. In this case a very small sample from the patient is required that is sufficient to yield a few micrograms of DNA. This is usually done by collecting blood sample and extracting DNA from leukocytes. The gene areas known to harbor mutations are amplified by use of the polymerase chain reaction (PCR). Depending on the type of the mutation, it is either detected directly by gel electrophoresis (e.g. trinucleotide repeat expansions, repeating gene sequences, etc.), digestion by a restriction enzyme followed by agarose gel electrophoresis (provided a particular mutation alters or creates a restriction site for some restriction enzyme) and direct amplicon (DNA amplification stretch) sequencing. However, if the gene size is very large, mutations may be spread throughout the gene stretch; direct mutational analysis will be time and labor expensive. In addition to this, a number of loci have been identified with PCG where genes involved are yet unknown, indirect molecular diagnosis can be done. However, it must be borne in mind that ‘indirect' molecular diagnosis helps in determination of risk of an individual to develop PCG if some other family member has already been diagnosed clinically or molecularly. This is brought about by a technique analyzing DNA polymorphisms (‘markers') known to be in close vicinity to the disease locus under investigation. What is important in this case is identification and development of the marker alleles in healthy and affected family members that will identify the disease gene-bearing chromosome in the family under consideration. This method comes with a caveat of a small but definite error rate. Until the gene identification by Sarfarazi (1994),^2,33^ predictive testing for PCG was the most widely used application for indirect molecular diagnosis. As more and more underlying genes were subsequently identified direct mutational analysis gained importance. Current molecular diagnostic methods for PCG are largely restricted to analysis of genomic DNA extracted from peripheral blood. But as new findings about the signal pathways involved, protein dysfunction and gene regulation come to forefront the following methods for genetic workout will become more and more important.

### Practical Approach to Molecular Diagnosis

**Fig. 5:**
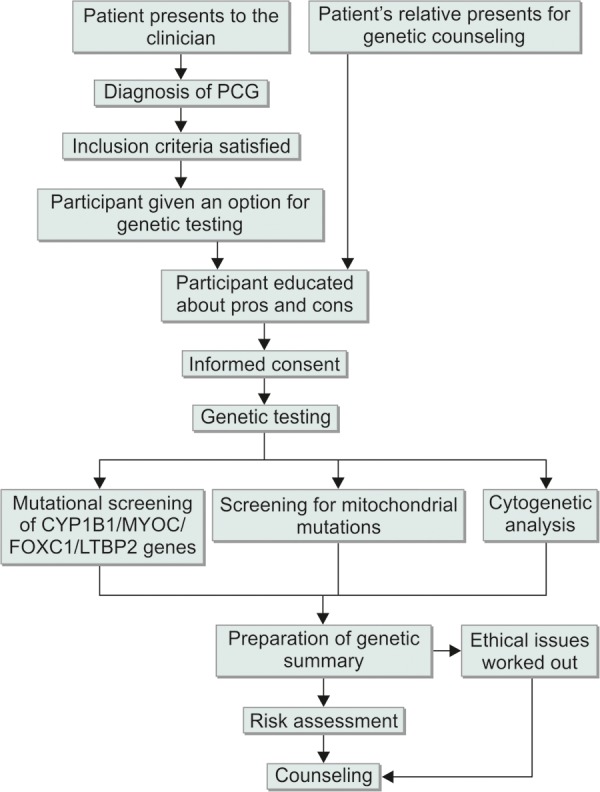
The procedure to be carried in genetic workout, diagnosis and counseling of PCG patients and/or individuals at risk

The procedural approach to molecular diagnosis of PCG has been outlined in Figure 5. This figure gives an overview of the approaches to genetic diagnostics and genetic counseling for both patient and/or a relative. The patient is clinically diagnosed or the relative of a PCG patient presents before the clinician or a genetic counselor. After resolving the potential ethical issues, the patient or participant (or legal guardian) signs on a written informed consent. Sample is then taken by well trained personnel. In most of the cases, the sample is peripheral blood drawn by venipuncture by a phlebotomist. A total of 1 to 10 ml of peripheral blood can be taken depending on various factors. EDTA can be used as an anticoagulant and the blood transported to the laboratory at room temperature (in case DNA is to be extracted). In case of RNA extraction, blood is immediately put on ice or minicooler and processed in the laboratory.

### Genotyping

Because many gene sequence changes in PCG either delete or create restriction sites (e.g. 376insA, 528G^A, 923C^T, 959G^A, 1449G^A and 1514C^A) for a wide range of commercially available restriction endonucleases; restriction fragment length polymorphisms (RFLP) is the first choice used for genetic mapping and diagnosis if done in combination with Southern blotting of genomic DNA.^44,45^ It is worth mentioning that RFLP is losing its importance with the advent of sequencing technology and various software tools that help in determining the pathological mutations in genetic disorders. However, there are still some remnant applications like mapping of large gene deletions for which Southern blotting still remains the best method.^46^ RFLP can be used to detect the most prevalent mutations in a population and special kits can be designed for rapid diagnosis.

### Amplification Refractory Mutation System

This method can be potentially used to detect already identified (or the most prevalent) mutations in PCG patients of a particular ethnicity. Though it is not currently in use but is a rapid and relatively cheap method. Though it is not a conclusive test but it has a high throughput value and can be reliably used in detection of mutation in nonaffected members of PCG families and those suffering from other genetic disorders. Amplification refractory mutation system (ARMS) is slight modification of conventional PCR. In this technique one of the primers is designed in a way so as to have a polymorphic base in the template at its 3’position.^47^ Then the usual PCR is carried out. The Taq polymerase is unable to perform polymerization from the mismatched base. In this way, the PCR product is formed only if the 3'base in the primer matches the template. It is imperative that it is a very low cost technique and can be employed, where funds are limited or the patient is not able to afford the cost of genetic testing.

### Single-Stranded Conformation Polymorphism

Single-Stranded conformational polymorphism (SSCP) employs some physical properties of single-stranded DNA. It can be used in diagnosis of a great number of PCG cases. The immense use of this method is that it is high throughput and cost effective. The principle underlying this technique is that single-stranded DNA folds in a sequence-specific manner in a nondenaturing solution and any slight change in DNA sequence precipitates a consequent change in this sequence-specific structure. Mutations alter these sequence-specific structures and the altered structure has different mobility compared to the original sequence structure in non-denaturing gel.^48^ Since there are a number of mutations that have been described in PCG, it is well effective to screen a large number of DNA sample by SSCP. SSCP is a technique with sensitivity between 35 and 100%. It must be borne in mind that SSCP is able to detect more than 80% of mutations. Alteration in the conditions of analysis increases its sensitivity significantly.^49,50^ However, DNA fragment size is a limiting factor in SSCP^51^ with drastic changes reported in sensitivity with DNA fragments more than 150 bases; 300 bases as the upper limit.^52^

### Heteroduplex Analysis and Conformation-Sensitive Gel Electrophoresis

Mutations in various genes in PCG and other genetic disorders can also be detected by heteroduplex analysis (HA) and conformation-sensitive gel electrophoresis (CSGE) in a nondenaturing gel. It is well known that heteroduplexes move slowly as compared to homoduplexes.^53^ This technique was originally described for insertion/deletion mutations, but in principle can be applied to detect single-base mismatches.^54^ Although there is evidence suggesting that sensitivity decreases in larger DNA fragments; HA has been successfully applied to detect mutations in DNA fragments with sizes greater than 1 kb.^55^ HA is an easy technique with no need of DNA labeling or any costly equipment.^56^ CSGE is a similar technique which employs slightly denturing conditions.^57^ HA and CSGE has a sensitivity of 88%.^58^

### Sequencing

Gene sequencing following PCR amplification with specially designed primers is currently the best used method for molecular diagnosis and characterization of PCG.^24,25^ In this method DNA is isolated from peripheral blood and subjected to PCR with specially designed primers to amplify the region of interest. The PCR product is purified and sequenced. The so obtained sequence is compared to the sequence (wild-type) already present in the various databases. An example, includes the 2001 Revised Cambridge Reference Sequence (http://infinity.gen.emory.edu/mitomap.html). The sequences can be aligned using the pubmed BLAST program (http://www.ncbi.nlm.nih.gov/blast/bl2seq/bl2.htlm). Once an evidence of sequence change is obtained (initially treated as polymorphism), it is confirmed manually. Data from patients with PCG is also compared with controls parallely enrolled in the study. If a sequence change is observed in both control as well as the patient, it is treated as polymorphism. If, however, sequence change in any gene of interest is observed in patients with PCG only, it is considered a new mutation which is confirmed by reamplification of the region using a separate sample for DNA extraction. Many novel mutations in PCG have been identified by this method only. We have also reported novel mutations in CYP1B1 gene using this method.^24,25^

### RNA Analysis

In many cases the mutations in a gene (or mutations in the regulatory regions which are not generally detected by sequencing) lead to changes in the expression pattern of the gene and often many other related genes. Mutation detection in introns and 3'- and 5'-untranslated regions (UTRs) or in promoters cannot be done by sequencing from the expressed gene. The problem becomes more relevant when promoters have not been characterized. However, if the mutation leads to change in expression of the gene, RNA analysis will help. In PCG, it is of particular importance because the main gene involved in its pathogenesis (CYP1B1) is very long. RNA analysis can be done by two methods. One is Northern blotting in which a radioactive probe is designed against the complimentary RNA regions and hybridization is achieved and the film is developed. The strength of the band on the film corresponds with expression levels in the gene. Another method is real-time PCR (RT-PCR) in which RNA is extracted from the patient sample and the first strand cDNA synthesis is carried out. Then the RT-PCR with specially designed primers is done. This method shows changes in expression in a variety of genes as compared to their normal expression. As and when literature is available about changes in the expression levels of CYP1B1 and other genes in PCG, RT-PCR will find its use in the diagnosis as well as prognosis in PCG.

### Cytogenetics

As many cytogenetic abnormalities have been reported in PCG like trisomy 8q22-qter, monosomy 9p23-qter, 22p+ variant chromosome, etc.^38-40^ karyotyping forms a good rationale to be carried out in patients where the representative genes do not harbor mutations.^41^ Cytogenetic analysis requires a peripheral blood sample (heparinized). The leukocytes are grown in eukaryotic cell medium and the cell division is arrested in the metaphase using colchicine. The cells are fixed and chromosomes are visualized under microscope and analyzed using relevant softwares (e.g. cytovision).

### Cloning and Protein Methods

Although these methods have not yet been employed in the detection and diagnosis in PCG but they are likely to be futuristic prognostic markers of the disease. Evaluation of the protein sequence variation and/or functional analysis of the protein of the gene of interest (CYP1B1, MYOC, etc. in this case) will give the overall efficiency of the metabolic pathways these genes are involved in. The functional assay of the protein products in question can help in tracing the prognosis of the disorder. Though protein methods are labor intensive and expensive but they provide information about the biological effects of gene mutations and in deciding pathogenecity of a particular mutation. In near future, these methods will form the bedrock of patient counseling. Along with other aspects; research in our laboratory is currently focusing on the functional genomics of PCG with evaluation of the effects of novel mutations on CYP1B1 gene function. A pathogenic mutation will predict the risk and penetrance of the disease while as a nonpathogenic mutation will have mild effect on vision. This will, in turn, affect the mode of patient management.

### The Protein Truncation Test

The protein truncation test (PTT), is a simple extrapolation of the detection of truncating mutations in the genes involved in a disease. This can very well be used in the detection of truncating mutations in genes involved in PCG. *In vitro* protein synthesis followed by SDS PAGE will detect the truncation. The advantage of this method is that it does not require expensive equipments like sequencer.^59,60^ The SDS PAGE picture of the protein of interest has a shift in size in case of truncated protein and can easily be detected by comparing it to the wild-type. This technique requires cDNA or large exons as a starting material. The biggest advantage of PTT is that only mutations with a functional consequence, such as truncating mutations, are identified.

### Functional Assays

There are some assays available (like EROD assay in evaluation of CYP1B1 protein function) which directly evaluate the protein function from a cloned DNA sequence.^61,62^ There are quite a few studies which have reported applications of functional assays^63^ in diagnostics. In this case, the patient's blood is taken and RNA is extracted. A cDNA is synthesized by reverse transcriptase PCR. The required gene is then cloned into a suitable vector and expressed in a suitable host. The so expressed protein is then evaluated with respect to various substrates. Its function is compared with that of the wild-type. This gives important knowledge about the extent of loss of function in the gene mutation and resultant effects. Since a functional assay is only possible if the function of the protein is known (which is known in case of CYP1B1) functional protein can be expressed *in vitro* and an assay designed.

**Figs 6A to D:**
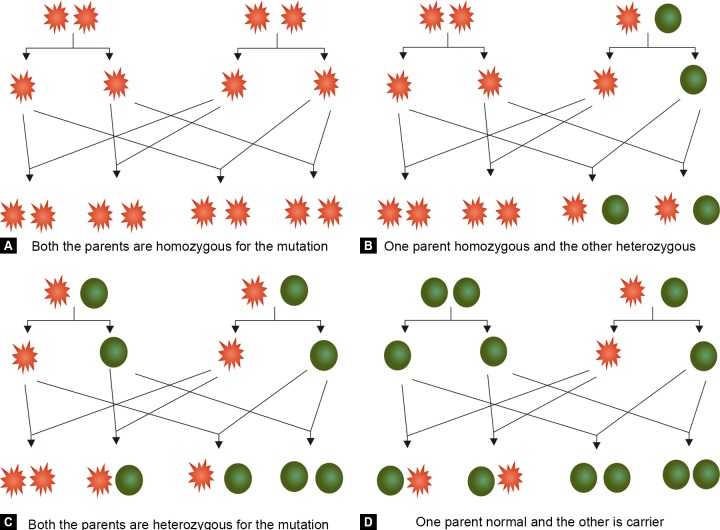
First row in each segment represents the parental genotype (spike = mutant; round = normal), second row represents the haploid gametes and the third row represents the first generation of offsprings

### Genetic Risk Assessment in PCG

PCG has varied patterns of inheritance and its etiology is yet unknown. Involvement of many genes in this disease makes its understanding rather complex. Currently very little is known about PCG genetics but as molecular mechanisms and patterns of inheritance are completely understood, genetic counseling will be necessary even in PCG management. Many PCG cases pose autosomal recessive inheritance but many sporadic cases also do occur. The frequency of gene mutations across many populations in the world is not known accurately. Differential penetrance (different genotype-phenotype correlation) in various mutations in same gene adds further momentum to the problem. There are some mutations which have a very severe phenotype while others may cause a very mild form of the disease. There may be some mutation which only brings down the expression level of the gene while others may arrest its function altogether. This fuzziness in the current understanding of PCG mechanism has kept clinicians and geneticists at bay in counseling PCG patients and/or their relatives. In spite of the problems mentioned above, genetic counseling can still be brought into existence in case of PCG. The chances of inheritance of a mutation by an offspring if the parents have the mutation can be estimated using the basic principles of genetics. Based on the possibilities that can bring about the disease from a mutation following conditions are possible. Figure 6 explains the following possibilities pictorially.

1. Both the parents are homozygous (both copies of the gene are mutated) for a mutation in the gene of interest— in this case the offspring will inherit the mutations in both gene copies with 100% possibility.
2. One parent is homozygous and another parent is heterozygous (only one copy of the gene is mutated) for mutation in the gene of interest—in that case there are 50% chances of each offspring being a carrier (heterozygous) and 50% chances of being homozygous for the mutation. No child with normal genotype will be born.
3. Both parents are heterozygous for the mutation in the gene of interest—in that case there are 25% chances for the offspring to be normal (without mutation in any copy of the gene), 50% chances of being the carrier and 25% chances of being homozygous for the mutation.
4. One parent is normal and the other parent is a carrier— in that case there are 50% chances for the offspring to be a carrier and 50% chances to be normal (without any mutation in any of the two gene copies). However, no child will be homozygous for this mutation.

It is to be noted that the chances of the inheritance pattern do not change with the number of siblings. The probability of having a particular mutation is independent of other siblings. However, the above discussion is for only a single mutation type in a single gene. If the mutation has a very low penetrance then heterozygous state may not produce disease in the patient but if the mutation is deleterious, the counselor needs to be very careful in advising marriage possibilities in such individuals.

Risk assessment is currently possible in case of mitochondrial mutations. If a correlation between a mitochondrial mutation and PCG is established, then it becomes very easy to predict the risk of developing the disease in the offspring. Since mitochondria are maternally inherited, the offspring will have a nearly 100% chance of inheriting the mutation if the mother harbors the mutation. In case the father's mitochondrial gene is mutated, there are negligible chances that the offspring will have the mutation.

Combining the above discussed features with genotyping finding, patients and their relatives can be educated and informed about various aspects of PCG and in many cases PCG can be prevented using proper counseling methods.

It is, therefore, imperative that every ophthalmic hospital should have a genetics unit which will help patients and their relatives to know if they carry the mutant or whether they are homozygous or heterozygous for the change.

### Genetic Counseling

Since molecular biology is seeing a rapid growth in deciphering etiology, pathogenesis and pathology of various disorders, genetic testing is likely to witness a great use in patient care. Genetic counseling is one of the important areas which exploit the use of molecular biology techniques in understanding many aspects of inheritable diseases. A proper understanding of the genotype-phenotype correlations and the modes of inheritance is necessary to counsel persons suffering from a particular inheritable disease and/or their relatives. Genetic counseling is important in knowing about the chance a second sibling contracts the disease. Genetic counseling is important in guiding marriages in order to get a favorable outcome. Genetic counseling is important because genetic testing enables us to establish the etiology of an inheritable disorder, predict probability of developing the disease, predict penetrance of the disease and predict chances of birth defects, offering ways to escape a bad outcome, suggest ways to escape the dangerous aspects of a particular disorder.^64^

It is mandatory that genetic testing should be followed by genetic counseling wherever possible. It will help the patient, carrier or family to plan for appropriate measures to prevent the disease. It is also likely to help various ethnic populations to understand the prevalence and necessary measures to prevent a genetic disorder. An example is consanguineous marriages which have lead to a great increase in prevalence of PCG in Slovakian gypsies, Saudi Arabian muslims and populations in Andhra Pradesh.^6-10^

**Figs 7:**
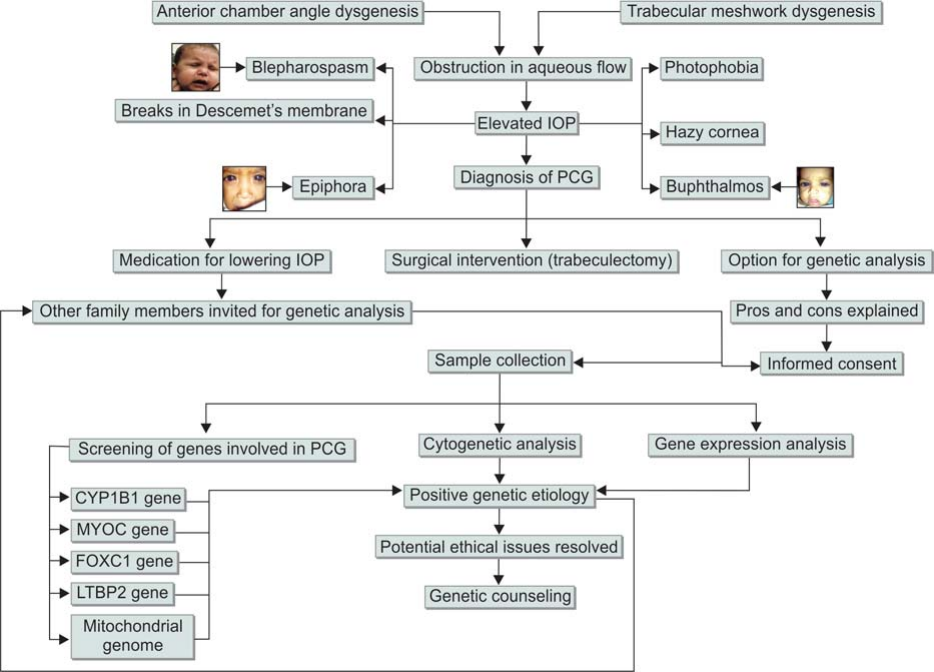
Overview of PCG with depictions of etiology, symptoms, clinical features, diagnosis, management, genetic evaluation and genetic counseling

Genetic testing for PCG is also likely to help in screening of large populations for this disorder so that occurrence of the disease is brought down substantially. Figure 7 illustrates the detailed overview of PCG management. It reveals the importance of genetic testing and counseling for patients and their family members. There is a need of genetic counseling unit in every hospital in which experts in genetics and medicine work in synergy to counsel patients for better management of their genetic conditions.

### Ethical Issues

The code of ethics of the National Society for Genetic Counseling (NSGC), United States of America clearly states that genetic counselors should ‘strive to enable their clients to make informed independent decisions, free of coercion, by providing or illuminating the necessary facts and clarifying alternatives and anticipated consequences'.^65^ Genetic evaluation reveals a great deal of knowledge about an individual which may be potentially harmful to the individual subjected to such tests. It may reveal some conditions and propensities about which the individual has no idea. Confidentiality of the records and well maintenance of the results are indispensable. A single false positive test can create a state of panic in any family which may even lead to legal issues. It is advisable that genetic scientists and counselors need to update themselves with the latest regulations and guidelines issued by various appellate authorities for genetic counseling in their respective countries.

## CONCLUSION

PCG is one of the most common causes of childhood blindness and its genetic etiology has been established. PCG patients and/or their relatives can be given an option for genetic evaluation in order to understand the disease well from research point of view as well as help the patient and/ or patient's relatives to take appropriate decisions regarding the disease. Molecular diagnostic approaches are likely to improve the diagnostic and management strategies of PCG dramatically over the next few decades. Genetic testing and molecular diagnostics have many new applications. It helps the clinician, patient and his/her family to take decisions for conditions not restricted to PCG only. Genetic testing aids in understanding the disorder thereby improving medical care, and the future is foreseen to yield continued advances in this direction. In the coming years, genetic counseling will be one of the important areas in PCG patient management.
